# A new option for early breast cancer patients previously irradiated for Hodgkin's disease: intraoperative radiotherapy with electrons (ELIOT)

**DOI:** 10.1186/bcr1310

**Published:** 2005-08-16

**Authors:** Mattia Intra, Oreste Gentilini, Paolo Veronesi, Mario Ciocca, Alberto Luini, Roberta Lazzari, Javier Soteldo, Gabriel Farante, Roberto Orecchia, Umberto Veronesi

**Affiliations:** 1 Department of Breast Surgery, European Institute of Oncology, Milan, Italy; 2 University of Milan, School of Medicine, Milan, Italy; 3 Medical Physics Unit, European Institute of Oncology, Milan, Italy; 4 Department of Radiotherapy, European Institute of Oncology, Milan, Italy

## Abstract

**Introduction:**

Patients who have undergone mantle radiotherapy for Hodgkin's disease (HD) are at increased risk of developing breast cancer. In such patients, breast conserving surgery (BCS) followed by breast irradiation is generally considered contraindicated owing to the high cumulative radiation dose. Mastectomy is therefore recommended as the first option treatment in these women.

**Methods:**

Six patients affected by early breast cancer previously treated with mantle radiation for HD underwent BCS associated with full-dose intraoperative radiotherapy with electrons (ELIOT).

**Results:**

A total dose of 21 Gy (prescribed at 90% isodose) in five cases and 17 Gy (at 100% isodose) in one case were delivered directly to the mammary gland without acute complications and with good cosmetic results. After an average of 30.8 months of follow up, no late sequelae were observed and the patients are free of disease.

**Conclusion:**

In patients previously irradiated for HD, ELIOT can avoid repeat irradiation of the whole breast, permit BCS and decrease the number of avoidable mastectomies.

## Introduction

The risk of developing breast cancer after radiotherapy for Hodgkin's disease (HD) is well documented and is attributed to the incidental inclusion of portions of the breast in the portals used to irradiate the mediastinum with or without infraclavicular/axillary regions [1,2]. This increased risk depends on several patient and treatment factors, including radiation dose, radiation treatment field and age at treatment [3]. The estimated risk is also affected by the duration of follow up after radiotherapy and the method used to calculate risk [4]. Based on concern about possible severe sequelae arising after a high total cumulative dose to the breast, several authors [5,6] have suggested mastectomy as the treatment of choice for breast cancer arising after radiotherapy for HD.

Few studies have been published whereby alternative therapies to mastectomy have been employed; they describe local excision alone, or interstitial brachytherapy and local excision followed by local field external beam radiotherapy [7-10].

In order to avoid a dangerously high total cumulative dose of radiotherapy to the whole breast, soft tissues of the thoracic wall, lung and heart, without rejecting the possibility of breast conserving surgery (BCS), we evaluated the potential of performing full-dose intraoperative radiotherapy with electrons (ELIOT) in women with early breast cancer who had been previously irradiated for HD.

## Materials and methods

Between July 1999 and April 2005, 756 breast cancer patients who were candidates for BCS received ELIOT as the sole radiation treatment in the European Institute of Oncology in Milan. Of these, 100 patients had been treated in the past within a preliminary validation phase I study [11,12] and 440 patients are currently in an institutional phase III ongoing randomized clinical trial, in which 21 Gy ELIOT (prescribed at 90% isodose) is prospectively compared with external postoperative fractionated radiotherapy (PFR) including elective treatment of the whole breast. Eligibility criteria for the ELIOT randomized trial included patients aged between 48 and 75 years affected by a unicentric breast invasive carcinoma with a maximum diameter of 2.5 cm. Locally advanced tumors (T3 and T4), the presence of a contralateral synchronous or metachronous tumor, non-invasive carcinomas (including Paget's disease), other breast malignancies different from carcinoma, multifocality or multicentricity of the disease, previous surgical biopsy and previous oncological history are considered exclusion criteria, while a breast thickness superior to 3 cm is considered a technical contraindication, it being impossible to deliver energy of more than 9 mega-electron volts (MeV) with the present ELIOT dedicated linac. No particular tumor breast locations are excluded from ELIOT, but the close proximity of the tumor to the skin and pectoral major muscle infiltration are considered relative contraindication for ELIOT. In fact, in these cases, the irradiation of the entire breast, including skin and pectoral major muscle, is mandatory. Finally, if the tumor is very close to the axilla, ELIOT is avoided due to the risk of irradiating the axillary nerves and vessels.

In a selected group of 216 invasive breast cancer patients who were candidates for BCS, and who had specifically asked for and signed informed consent for ELIOT or in whom PFR was not considered safe or feasible due to clinical reasons (e.g. severe cardiopathy, large scars from skin burns, vitiligo, geographic or social obstacles), ELIOT was performed outside the randomized trial. In this selected group of patients, only the tumor size (larger than 2.5 cm), locally advanced disease, multifocality or multicentricity were considered exclusion criteria. Of these 216 patients, 6 were previously treated for HD and received mantle radiation (Table 1).

The breast cancer presentation of these six patients is described in Table 2. Five patients underwent BCS and radioguided sentinel lymph node biopsy according to the standard technique adopted at the European Institute of Oncology [13]. In four out of the five cases, the biopsy showed that the sentinel lymph nodes were free of metastasis, so complete axillary dissection was not performed. In one patient, due to the presence of clinically metastatic axillary nodes, a complete axillary dissection was performed directly.

The radio-surgical technique of the six ELIOT procedures did not differ from the 'classic' technique, which has been described previously [14] in patients not previously irradiated for HD.

To evaluate acute and late radiation morbidity, the Radiation Therapy Oncology Group (RTOG) and European Organization for Research and Treatment of Cancer (EORTC) scoring scheme [15] was applied on the first and seventh day of the radio-surgical treatment and every six months during follow up.

### Radiation therapy device

The dedicated ELIOT machine (Novac7, Hitesys, Italy) is a miniaturized linear accelerator that can be moved by a robotic arm, docked in the operating room and fulfils the statutory requirements with respect to radiological protection. Additional barriers (from 5 to 15 mm lead) are provided for positioning around the operating table. A lead shield (15 cm thick) is also placed under the surgical bed corresponding to the electron field (beam stopper). ELIOT delivers a single full-dose of radiation directly to the tumor bed after removal of the tumor. Based on the radiobiological models used to predict radiation effects (linear-quadratic surviving fraction or multitarget surviving fraction) [16], we can estimate that a dose of 60 Gy delivered at 2 Gy daily, which is the radiation dose required to control the microscopical residual disease after breast resection, is equivalent to a single fraction of 20 to 22 Gy when using an α/β ratio at 10 Gy, typical for tumors and acute reacting tissues. Using the same equation, but calculating the tolerance of late responding tissues (α/β ratio at 3 Gy), this equivalent value increases to at least 110 Gy. The linac does not produce photons, but only delivers electron beams at four different energy levels: 3, 5, 7 and 9 MeV as nominal energies, corresponding to 4.5, 5.2, 6.5 and 7.8 MeV effective energies to the phantom surface, respectively. The depth of 80% isodose ranged between 13 mm (3 MeV, not used in clinical practice) and 24 mm (9 MeV).

In one patient, a total dose of 17 Gy (prescribed at 100% isodose) using electron beams (7 MeV energy) was delivered. In five patients, 21 Gy (prescribed at 90% isodose, 7 or 9 MeV of energy) were administered.

## Results

The biological characteristics of the tumors are shown in Table 3. Two patients received adjuvant chemotherapy with cyclophosphamide-methotrexate/5-fluorouracil, followed by tamoxifen 20 mg/day for 5 years associated in one case with LHRH analogue for 2 years. One patient received adriamicin/cyclophosphamide followed by tamoxifen 20 mg/day for 5 years associated with LHRH analogue for 2 years. Three patients received tamoxifen 20 mg/day for 5 years, associated in two cases with LHRH analogue for 2 years.

ELIOT was well tolerated in all patients without any unusual acute reactions (grade 0 according to the EORTC/RTOG acute radiation morbidity criteria). In the patients with longer follow up, no late sequelae were observed (grade 0 according to the EORTC/RTOG late radiation morbidity scoring scheme) and all women had an excellent cosmetic result. All the patients are free of disease after 53, 38, 29, 24, 23 and 17 months of follow up (average 30.8).

## Discussion

Previous mantle radiotherapy for HD is considered a contraindication to BCS and radiotherapy [17]. This is based on concerns about possible severe sequelae arising from a high total cumulative dose, exceeding normal tissue tolerance, being delivered to the portions of the breast that have presumably already received radiation for the lymphoma, even though modifications of the breast gland over time render the exact calculation of the dose infeasible many years after radiation delivery. Most authors consider these patients at significant risk of complications (fibrosis, skin and soft tissue necrosis, rib fractures, potential lung and heart toxicities) [6] and do not candidate them for BCS and adjuvant radiotherapy. In contrast, other reports [18,19] support BCS followed by PFR when breast cancer develops many years after radiotherapy for HD. At present, no consensus exists regarding the correct management of breast cancer after mantle irradiation for HD and, given the discordant results and the small number of women treated with BCS, mastectomy continues to be recommended as the standard treatment. To avoid a high total cumulative dose to portions of the breast or soft tissues of the thoracic wall, one of the conservative options is to treat just the tumor bed: the irradiation of a small volume of the breast and adjacent structures could allow the risk of complications to be minimized [7-10]. Therefore, over the past decade there has been increasing interest in a variety of radiation techniques designed to treat only the portion of the breast deemed to be at high risk for local recurrence [20]: these include brachytherapy implants, MammoSite applicator, intraoperative orthovoltage device, and 3D conformal or intensity-modulated external radiotherapy (partial-breast irradiation (PBI)). All these techniques have similar indications but different applications [21,22]. In particular, they differ in the source of radiation (for example, X-ray, Iridium, photons) and the amount of breast volume treated. Although 5 to 7 year outcome data on patients treated with PBI are now becoming available, many issues remain unresolved, including the clinical and pathological selection criteria of patients, radiation dose and fractionation, and how these relate to the standard fractionation for whole breast irradiation, appropriate target volume, local control within the untreated ipsilateral breast tissue, and overall survival. With a view to furnishing guidelines and clarifying the issues of major controversy, a workshop on PBI was held in Bethesda, in December 2002. The workshop report emphasized the importance of education and training with regard to the results of PBI as it becomes an emerging clinical treatment [23]. In particular, ELIOT, a new radiotherapeutic technique that delivers a single dose of radiation directly to the tumor bed during the conservative surgical treatment of early breast cancer, has been proposed for evaluation in randomized clinical trials as a possible alternative to standard PFR.

When in July 1999 we focused our interest on the use of intraoperative radiotherapy as an exclusive treatment in small unifocal infiltrating breast carcinomas, we considered that ELIOT could be specifically applied in all those situations in which PFR was not considered safe or feasible for various reasons (for example, severe cardiopathy, large hypertrophic scarring from skin burns, vitiligo, geographic or social obstacles) [24] and, in particular, in patients irradiated for HD. After our first preliminary report [25], ELIOT was proposed in six patients previously irradiated for HD. In all cases, the dose delivered and the energy of electron beams from ELIOT did not differ from those in the other 549 patients submitted to ELIOT; in one patient a total dose of 17 Gy (prescribed at 100% isodose) using electron beams at 7 MeV energy and in five patients doses of 21 Gy (90% isodose, 7 and 9 MeV of energy) were delivered. The different electron energies administrated (7 or 9 MeV) are related to the different breast gland thickness.

The radio-surgical technique of the six ELIOT procedures did not differ from the 'classic' technique used for previously non-irradiated patients [14]. In particular, to ensure a good coverage of the target by the radiation dose and maximal protection of the normal tissues in the operative area, adequate preparation for irradiation of the portion of breast gland to undergo ELIOT is necessary. Protective devices are placed between the gland and the pectoral muscle; a dedicated lead disk 5 mm thick and an aluminium disk 4 mm thick, available in various diameters (4, 5, 6, 8, 10 cm) are commonly used. The wall protection is guaranteed both by the absorption properties of the lead and aluminium and the 9 mm outdistance created by the disks.

We scored the radiation morbidity according to the RTOG/EORTC criteria [15]. ELIOT was well tolerated without any unusual acute reactions despite previous breast irradiation. We did not observe any ischemic or necrotic problems of the skin flap due to the careful sparing of the subcutaneous vessels during the mobilization of the residual breast around the tumor bed. No increased post-operative complications (pain, seroma, emathoma, infection) were observed in these six patients when compared to the overall group of ELIOT patients. The length of hospital stay was therefore not prolonged. The cosmetic outcome was also very good in all patients: no skin erythema was observed as a result of the complete removal of the skin from the radiation beam.

The follow up is too short (average 30.8 months) to evaluate late sequelae in these six re-irradiated patients but no complications were observed in the four patients with more than 2 years of follow up.

## Conclusion

ELIOT dramatically reduces the radiation exposure of the normal tissues and, in particular, of the previously irradiated breast, avoiding a high total cumulative dose to the gland and to the soft tissues of the thoracic wall. In patients previously treated for HD, ELIOT permits BCS independently of the interval between mantle radiotherapy and breast surgery, without acute local complications, decreasing the number of avoidable mastectomies. A longer follow up is necessary to evaluate the incidence of radiation-induced sequelae and the local recurrence rate.

## Abbreviations

BCS = breast conserving surgery; ELIOT = intraoperative radiotherapy with electrons; EORTC = European Organization for Research and Treatment of Cancer; HD = Hodgkin's disease; MeV = mega-electron volts; PBI = partial-breast irradiation;PFR = postoperative fractionated radiotherapy; RTOG = Radiation Therapy Oncology Group.

## Competing interests

The author(s) declare that they have no competing interests.

## Authors' contributions

MI conceived the study, and participated in its design and coordination and drafted the manuscript, OG helped to draft the manuscript, PV participated in the coordination of the study, MC participated in the coordination of the study, AL participated in the design of the study, JS carried out patient follow up, GF carried out patient follow up, and RO and UV participated in the design of the study. All authors read and approved the final manuscript.

## Figures and Tables

**Table 1:** Presentation of and therapy for Hodgkin's disease

Patient no.	Age (years)	Diagnosis	Stage	Therapy
1	29	HD, MC	IIA	MOPP × 6, Mantle 36 Gy
2	28	HD, NS	IIB	MOPP × 4, ABVD × 4, Mantle 20 Gy
3	42	Had	IIB	ABVD × 3, Mantle 40 Gy
4	38	HD, MC	IIA	MOPP × 6, Mantle 40 Gy
5	24	HD, NS	IIB	MOPP × 6, Mantle 40 Gy
6	30	HD, NS	IIA	MOPP × 4, ABVD × 4, Mantle 36 Gy

^a^No histologic subtype determined. ABVD, adriamicine, bleomycin, vinblastine, dacarbazine; HD, Hodgkin's disease; MC, mixed cellularity; MOPP, nitrogen mustard, vincristine, procarbazine, prednisone; NS, nodular sclerosis.

**Table 2:** Presentation of breast cancer

Patient no.	Age (years)	Interval since radiation for HD (years)	ELIOT dose
1	35	16	21 Gy (90% isodose), 7 MeV
2	42	14	17 Gy (100% isodose), 7 MeV
3	50	8	21 Gy (90% isodose), 9 MeV
4	50	12	21 Gy (90% isodose), 9 MeV
5	34	10	21 Gy (90% isodose), 7 MeV
6	39	9	21 Gy (90% isodose), 9 MeV

HD, Hodgkin's disease; MeV, mega electron volts.

**Table 3:** Characteristics of the breast cancer

Patient no.	Tumor site	T	N	G	ER, PgR	KI67	PVI
1	UO	1c	0 (sn)	2	15%, 25%	11%	No
2	LI	1c	0 (sn)	3	0%, 0%	70%	No
3	C	1c (is)	0 (sn)	1	90%, 90%	13%	No
4	S	1c	1a	2	85%, 85%	25%	No
5	UO	2	1a	2	30%, 0%	27%	Yes
6	UO	1b	0(sn)	1	90%, 90%	8%	No

T, tumor size; N, axillary lymph nodes; G, tumor grade; ER, estrogen receptors; PgR, progesterone receptors; KI67, proliferative index; C, central; is, extended intraductal component associated; LI, lower inner; PVI, perivascular invasion; S, superior; sn, sentinel node; UO, upper outer.
